# *Hechtia glomerata* Zucc: Phytochemistry and Activity of Its Extracts and Major Constituents Against Resistant Bacteria

**DOI:** 10.3390/molecules24193434

**Published:** 2019-09-21

**Authors:** Tommaso Stefani, Elvira Garza-González, Verónica M. Rivas-Galindo, María Yolanda Rios, Laura Alvarez, María del Rayo Camacho-Corona

**Affiliations:** 1 Universidad Autónoma de Nuevo León, Facultad de Ciencias Químicas, Av. Universidad S/N, Ciudad Universitaria, CP San Nicolás de los Garza 66451, Nuevo León, Mexico; tstefani854@gmail.com; 2 Universidad Autónoma de Nuevo León, Servicio de Gastroenterología Hospital Universitario Dr. José Eleuterio González, Av. Gonzalitos y Madero S/N, Col. Mitras Centro, CP Monterrey 64460, Nuevo León, Mexico; elvira_garza_gzz@yahoo.com; 3 Universidad Autónoma de Nuevo León, Facultad de Medicina, Av. Madero S/N, Col. Mitras Centro, CP Monterrey 64460, Nuevo León, Mexico; veronica.rivasgl@uanl.edu.mx; 4 Universidad Autónoma del Estado de Morelos, Centro de Investigaciones Químicas IICBA, Av. Universidad 1001, Col. Chamilpa, Cuernavaca, Morelos 62209, Mexico; myolanda@uaem.mx (M.Y.R.); lalvarez@uaem.mx (L.A.)

**Keywords:** infections, multi-drug resistant, phytochemistry, traditional medicine, northern America

## Abstract

*Hechtia glomerata* Zucc. is used both as a source of food and in ethnomedicine to treat various diseases derived from bacterial infections such as bronchitis, laryngitis, nephritis, whooping cough, urethritis, and sepsis. There are no previous reports about its chemistry and biological activities. Therefore, the aims of this study were to identify components from organic and aqueous extracts of *H. glomerata* and test the extracts and major isolate compounds against resistant bacteria. Hexane, CHCl_3_/MeOH, and aqueous extracts were prepared and analyzed by different chromatographic techniques. Structural elucidation was carried out by NMR spectroscopy and X-ray diffraction. The antibacterial activities of extracts, phytochemicals, and semisynthetic derivatives against resistant bacteria were determined by the broth micro-dilution method. From the hexane extract nonacosane (1), hexatriacontanyl stearate (2), hexacosanol (3), oleic acid (4), and β-sitosterol (5) were isolated and characterized. From the CHCl_3_/MeOH extract, *p*-coumaric acid (6), margaric acid (7), caffeic acid (8), daucosterol (9), and potassium chloride (10) were isolated and characterized. A total of 58 volatile compounds were identified by GC-MS from the hexane extract and two solids were isolated from the CHCl_3_/MeOH extract. The UPLC-QTOF-MS analysis of the aqueous extract allowed the identification of 55 polar compounds. Hexane and aqueous extracts showed antibacterial activity against ESBL *Escherichia coli*, and three strains of *Klebsiella pneumoniae* ESBL, NDM-1 +, and OXA-48 with MIC values of 500 µg/mL. The CHCl_3_/MeOH extract was devoid of activity. The activity of phytocompounds and their semisynthetic derivatives toward resistant bacteria was weak. The most active compound was β-sitosterol acetate, with a MIC value of 100 µg/mL against carbapenem-resistant *A. baumannii*. This is the first report of the secondary metabolites of *H. glomerata* Zucc. and the activity of its extracts and major pure compounds against resistant bacterial strains.

## 1. Introduction

Infections by resistant bacteria represent a constant threat to human health and are becoming more difficult to treat each year [1]. In fact, the world is on the verge of a global crisis partially generated by the misuse of antibiotic agents and due to the unavailability of new drugs [2]. Drug resistant (DR), multi-drug resistant (MDR), and extremely drug resistant (XDR) bacterial infections are assumed to be responsible for thousands of human deaths every year and it is estimated that they will cause 10 million extra deaths worldwide by 2050 [3]. Thus, comprehensive efforts are necessary to reduce bacterial drug resistance, and to support the research and development of new effective drugs. The World Health Organization (WHO) published a list of bacterial strains with the greatest need of new antibiotics in 2017, prioritizing them based on the gravity of resistance as medium, high, or critical [4]. For these reasons, the discovery of new drugs is of utmost importance to control DR, MDR, and XDR bacterial infections.

Nature has always been a source of new and complex molecules and plant natural products have already proven useful in pharmaceutical chemistry [5]. The diversity of structures and biological activities of phytochemicals makes them very appealing from a new drug research and design point of view. Mexican medicinal plants are often used by the indigenous population to treat cancer and infections, among other diseases [6,7]. This knowledge makes them an interesting group of candidates for the research of new bioactive molecules that could be used directly or as lead compounds to treat bacterial infections.

In this work, we investigate the plant *Hechtia glomerata* Zucc., also known by the Mexican and Latino-American population as Guapilla or Lechuguilla. This plant is native to Guatemala, Mexico, and the southern regions of the United States, and is part of the family Bromeliaceae (56 genera and over 3000 species) [8]. Because of its vast distribution, it is considered today as the Bromeliad with the largest habitat [9]. In Mexico, there are 426 species of Bromeliaceae [10] and some are widely known, such as pineapple (*Ananas comosus*), and others have already been studied for pharmacological purposes [11,12,13,14,15,16] and part of their composition has already been elucidated [17,18]. *H. glomerata* is used both as a source of food and in ethnomedicine to treat various infections including bronchitis, laryngitis, nephritis, whooping cough, urethritis, and sepsis [19,20,21,22]. There are no chemical and pharmacological studies of *H. glomerata*. Therefore, a conventional phytochemical study of this plant was carried out. The extracts and pure isolated compounds were tested against resistant bacteria because it could be a source of antibacterial agents.

## 2. Results and Discussion

### 2.1. Isolated and Derivatized Compounds from the Hexane and CHCl_3_/MeOH Extracts

Five compounds were isolated and characterized from the Hex extract: nonacosane (1), hexatriacontanyl stearate (2), hexacosanol (3), oleic acid (4), and β-sitosterol (5). All are constituents of the vegetal cuticle. More specifically, compounds 1-4 are part of the waxes that cover the aerial part of plants [23], and perform a protective function, which prevents dehydration in arid environments [24]. A further five compounds were isolated and characterized from the CHCl_3_/MeOH extract: *p*-coumaric acid (6), margaric acid (7), caffeic acid (8), daucosterol (9), and potassium chloride (10). Compound 10 is an inorganic salt needed by halophyte plants to maintain homeostasis in their tissues [25]. Compound 9 is an abundant monoglycosylic saponin derivative of the aglycone 5, which is also in a high concentration in the Hex extract. Compounds 6 and 8 are abundant in cereals and grasses. These compounds have the most varied functions in plants and fungi and are precursors to different polyphenolic compounds. In particular, *p*-coumaric acid has a central role in the metabolism of these compounds and is the precursor of other phenolic compounds such as flavonoids, coumarins, lignans, lignin, and other secondary metabolites [26,27]. Some of the compounds (4, 5, and 9) were derivatized in order to improve their structural characterization and to investigate how their biological activity could change. Compounds 5 and 9 were acetylated, while compound 4 was methylated, giving the following derivatives: β-sitosteryl acetate (5a), daucosteryl tetraacetate (9a), and methyl oleate (4m).

### 2.2. GC-MS Analysis of Hexane Extract and Two Solids Obtained from CHCl_3_/MeOH Extract

A total of 49 compounds were identified from the Hex extract using Gas Chromatography Mass Spectrometry (GC-MS): 16 hydrocarbons (16.92%), 15 fatty acids (FAs, 37.04%), six steroids (31.46%), four terpenoids (4.65%), three long chain alcohols (3.11%), three aldehydes (0.35%), and two unknown compounds (6.47%). The GC-MS analysis of two mixtures of lipophilic compounds (HG1 and HG2) isolated from the CHCl_3_/MeOH extract showed 17 volatile compounds. Ten of these compounds were also found in the Hex extract, while the others were only present in the HG1 and HG2 samples. A total of 58 volatile compounds were identified in the Hex extract and solids HG1 and HG2 (see Table 1).

If we compare the constituents of Hex extract and the solids HG1 and HG2, we can see quantitative and qualitative differences. The Hex extract was mainly composed of fatty acids (FAs) and their derivatives including: eleven FAs, three FA esters, three fatty alcohols, and three aldehydes. The FAs were made up of a higher quantity of unsaturated fatty acids (UFAs, 18.66%) than saturated fatty acids (SAFAs, 11.20%).

Fatty alcohols are derivatives of the reduction of FAs and are involved in plant biosynthesis of esters and waxes [23]. The three aldehydes known as octanal (0.09%), nonanal (0.11%), and 2-(*E*)-decenal (0.15%) can be obtained from oxidation of fatty alcohols or reduction of FAs [28]. The second most abundant group of compounds was the hydrocarbons group including alkanes (15.56%) and alkenes (1.36%). The alkanes can be derived by decarboxylation of long chain FAs [29]. As mentioned before, hydrocarbons and fatty acids are the main constituents of plant cuticles, which explain why they are so abundant in the extract.

The rest of the compounds were terpenoids and steroids. Among the terpenoids were phytol (1.72%) and its derivative phytone (2.65%). Other terpenoid compounds identified were trachylobane (0.11%) and kaur-16-ene (0.16%). These trachylobane and kaurane diterpenoids are considered to be intermediates in the biosynthesis of the plant growth hormones gibberellins [30]. Terpenoids and steroids are also part of the cuticle. Among the sterols were β-sitosterol (17.97%), campesterol (5.38%), and stigmasterol (1.37%), which are very common in plants [31]. Lastly, the volatile compounds identified in the solids HG1 and HG2 were 12 alkanes (82.96%), four alkenes (6.73%), and two fatty alcohols (9.04%).

### 2.3. UPLC-QTOF-MS Analysis of the Aqueous Extract

Fifty-five compounds were identified from the ultra-high performance liquid chromatography quadrupole time-of-flight mass spectrometry (UPLC-QTOF-MS) analysis of the aqueous extract (Table 2). These compounds included 30 flavonoids (55.21%), nine phenolic compounds (15.80%), five saponins (9.43%), three terpenoids (5.46%), two stilbenes (3.97%), one nitrogen-containing compound (N-compounds, 2.10%), and five unknown compounds (8.03%). The major identified compounds were flavonoids derivatized with one or more sugar moieties. Some of these compounds were anthocyanins and anthocyanidins (21.06%), which are plant pigments already reported in the Bromeliaceae family [32]. One of the identified compounds was ananaflavoside B, previously identified in *Ananas comosus* [33].

Phenolic compounds were also identified from the aqueous extract including isochlorogenic acid (2.06%) and aloesol 7-glucoside (1.62%). Aloesol is a chromone, which is produced by the same pathway as flavonoids and coumarins. Aloesol has never been reported in the Bromeliaceae family, but was previously reported as a constituent of *Aloe vera* (Liliaceae) [34]. Chromones, much like flavonoids, have antioxidant activity and provide a defense against pests. Furthermore, chromones also have antifungal activity [35]. Other compounds identified include the steroidal saponins: daucosterol (2.12%) and agavoside B (1.44%), stilbenes: piceid (2.45%) and piceatannol (1.51%), terpenoids: ergosterol endoperoxide (1.97%), and zeaxanthin (1.47%), and the N-compound, guanosine pentaphosphate.

### 2.4. Antibacterial Activity

Extracts and major pure compounds were evaluated for their activity against resistant bacteria. Table 3 shows that the Hex extract and the aqueous extract has antibacterial activity against *E. coli* extended spectrum β-lactamase (ESBL), and three strains of *K. pneumoniae*: oxacillin, ESBL, and New Delhi metallo-β-lactamase 1 (NDM-1), with a minimum inhibitory concentration (MIC) value of 500 µg/mL. The CHCl_3_/MeOH extract showed null activity against all resistant strains tested. It is possible that the activity of the CHCl_3_/MeOH extract against resistant bacteria was masked or inhibited by other compounds present in this extract.

The antibacterial properties of the Hex extract may be linked with the good quantity of FAs contain in it, especially unsaturated fatty acids (UFAs) such as linoleic acid. Some FAs have been reported to have antibacterial activities against different strains of bacteria and mycobacteria. The mechanism of action involved in their antibacterial activity has been shown to involve the inhibition of the bacterial enzyme enoyl-acyl carrier protein reductase (FabI) [36]. It has been reported that phytol has activity against eight bacterial and eight fungal strains, which show a strong antibacterial effect with MICs between 3 and 38 µg/mL against *Bacillus cereus*, *Micrococcus luteus*, *Listeria monocytogenes*, *P. aeruginosa*, *Salmonella typhimurium*, *E. coli*, *Enterobacter cloacae*, and *Micrococcus flavus* [37]. In other studies, sub-lethal concentrations (0.5, 0.25, and 0.125 MIC) of phytol have shown anti-quorum sensing on *P. aeruginosa* [38]. Pythone is also believed to be an antibacterial agent, which has been reported as part of some plant essential oils that displayed antibacterial activity against sensitive bacteria [39]. The diterpenoids trachylobane and kaur-16-ene are also reported to have antibacterial activity [40].

Regarding stigmasterol and campesterol, few reports have tested these sterols as pure compounds, but the biological activity of their mixtures and derivatives is well reported. For example, their acetylated derivatives have shown antibacterial activity against *S. aureus*, *E. coli*, *P. aeruginosa*, and *Klebsiella* [41]. β-Sitosterol and daucosterol, which are in high concentration in the organic extracts of *H. glomerata*, are reported to have no antibacterial properties against *E. coli*, *S. aureus*, and *K. pneumonie* (MIC > 500 µg/mL) [42].

The activity of the aqueous extract is also partially explained by its composition. The high content of flavonoids is likely the main reason for the antibacterial activity of this extract [43]. Luteolin and kaempferol were found as glycoside derivatives in the extract. Kaempferol has been reported as active against methicillin-resistant *S. aureus* (MRSA), vancomycin-resistant *enterococci* (VRE), and *Burkholderia cepacia*, with MICs of 512 µg/mL. Luteolin was active against *E. coli*, *K. pneumoniae*, and *P. aeruginosa* (ATCC) presenting MICs of 125 µg/mL for all strains [44]. Other molecules with antimicrobial activity include chromones such as aloesol and its derivatives, which are being studied as mushroom tyrosinase inhibitors [35]. The saponins identified in the aqueous extract might also account as part of its activity against the resistant bacteria. For example, hederagenin was tested against three strains of *S. aureus* (ATCC 6538p, 25923, 29213) and various Gram-negative strains comprising *P. aeruginosa* (ATCC 27853), *A. baumannii* (ATCC 19606), and *E. coli* (ATCC 25922, 8739). This saponin was active (MIC 400–500 µg/mL) against the Gram-positive bacteria, whereas the Gram-negative strains were unaffected at any concentration [45]. Triterpene saponins of oleanolic acid have also been shown to have antibacterial activity against *S. aureus*, *P. aeruginosa*, and *K. pneumoniae*, with MICs ranging from 3.125 to 6.25 µg/mL [46]. Stilbenes have been reported to inhibit the electron transport chain (ETC), the cleavage of deoxyribonucleic acid (DNA), and cell division, and also shown to have antibiofilm activity [47]. The stilbene piceid is the main glycosylated derivative of resveratrol, and had activity against foodborne pathogens including *S. aureus* and *E. coli* with a MIC value ranging from 0.625 to 521 µg/mL against the Gram-positive bacteria, and, for Gram-negative bacteria, ranging from 16.5 to 260 µg/mL [48]. In another study, resveratrol was tested against *S. aureus* (ATCC 25923), methicillin-sensitive *S. aureus* (MSSA), MRSA, *E. coli* (ATCC 25922), *E. coli* (clinical isolate), *K. pneumoniae* (ATCC 13883), *K. pneumoniae* (clinical isolate), *P. aeruginosa* (ATCC 27853), and *P. aeruginosa* (clinical isolate). For the Gram-positive bacteria, the MIC value range was from 50 to 200 µg/mL, while, for the Gram-negative bacteria, MICs were higher than 400 µg/mL [49]. Piceatannol was reported to have inhibitory activity against some bacterial strains obtained from the National Collection of Type Culture (NCTC) like *S. aureus* (NCTC 6571) and *E. coli* (NCTC 10418) with MICs of 25 and 50 µg/mL respectively, while no effect was reported for *P. aeruginosa* (>1000 µg/mL) [50].

In our study, two of the most abundant isolated compounds and their derivatives were tested for antibacterial activity (Table 3). β-Sitosterol showed weak activity (MIC 200 µg/mL) against linezolid-resistant *S. epidermidis*, vancomycin-resistant *E. faecium*, carbapenem-resistant *A. baumannii*, carbapenem-resistant *P. aeruginosa*, and NDM-1 *K. pneumoniae*. β-Sitosterol acetate displayed weak activity (MIC 200 µg/mL) toward linezolid-resistant *S. epidermidis*, vancomycin-resistant *E. faecium*, carbapenem-resistant *P. aeruginosa*, ESBL *E. coli*, and ESBL *K. pneumoniae*, and moderate activity against carbapenem-resistant *A. baumannii* (MIC 100 µg/mL). Daucosterol showed weak activity (MIC 200 µg/mL) against linezolid-resistant *S. epidermidis*, vancomycin-resistant *E. faecium*, carbapenem-resistant *A. baumannii*, and carbapenem-resistant *P. aeruginosa*. Daucosteryl tetracetate had weak activity (MIC 200 µg/mL) toward vancomycin-resistant *E. faecium*, carbapenem-resistant *A. baumanii*, and carbapenem-resistant *P. aeruginosa*. The activity of the phytocompounds and their semisynthetic derivatives toward resistant bacteria was weak. The most active compound was β-sitosteryl acetate, with a MIC of 100 µg/mL against carbapenem-resistant *A. baumannii*. It has been reported that this compound had some activity on the growth of various bacterial strains, such as *S. aureus*, *E. coli*, and *K. pneumoniae* [51]. Daucosterol and its tetraacetate derivative gave almost the same results of antibacterial activity. These molecules are larger than their aglycones. Thus, they are not able to reach the cytoplasm and exert their effect on bacteria. In our study, *p*-coumaric acid did not have any activity at the highest concentration, whereas caffeic acid inhibited the growth of two Gram-positive resistant bacterial strains *S. epidermidis* and *E. faecium* (MICs: 200 µg/mL). Reports in the literature about the antibacterial activity of *p*-coumaric acid are contradictory. Lou et al. found that this compound inhibited Gram-positive bacteria such as *S. aureus* (MIC: 20 µg/mL) and Gram-negative bacteria *E. coli* (MIC: 80 µg/mL). It was also reported that *p*-coumaric acid has a double damage mechanism of action comprising an increase in membrane permeability and binding on the phosphate anion of bacterial DNA [52]. In other works, the activity of this compound against *S. aureus* has been reported as much higher (MIC: 625 µg/mL) or completely absent for both *p*-coumaric acid and caffeic acid (MIC > 1 mg/mL) against *S. aureus*, *S. epidermidis*, and *E. coli* [53]. Therefore, the inactivity of *p*-coumaric acid and caffeic acid against the tested Gram-negative strains and the inactivity of *p*-coumaric acid against the Gram-positive strains might be a result of the drug resistance of the tested bacteria.

From the above results, the activity of *H. glomerata* against resistant bacteria can be partly explained. However, it will be necessary to test the above extracts against sensitive bacteria strains and carry out a bio-assay guided study on the active extracts to isolate their active compounds in order to explain the use of this plant in Mexican traditional medicine. Furthermore, preparing other semisynthetic analogues of the most active compound should be considered to do a structure-activity relationship (SAR) study and improve its antibacterial activity.

## 3. Materials and Methods

### 3.1. General

The chemicals and reagents were acquired commercially from Sigma-Aldrich and Baker. Melting points (mp) were measured with a Thermo Scientific P12144Q Fisher-Johns Melting Point Apparatus (Thermo Fisher Scientific, Waltham, MA, USA). IR spectra were recorded with a Bruker ALPHA ATR-FTIR spectrometer (Bruker, Billerica, MA, USA). The 1D and 2D NMR spectra were obtained on a Bruker NMR Avance IIIHD spectrometer (Bruker Biospin, Billerica, MA, USA) operating at 400 and 100 MHz, respectively, for ^1^H and ^13^C measurements. For the ^13^C NMR spectra, multiplicities were determined by DEPT experiments (90° and 135°). X-ray diffraction (XRD) data were obtained in a Bruker SMART APEX and a Bruker-D2 Phaser (Bruker, Billerica, MA, USA). Normal phase TLCs were on Silica gel 60 F254 precoated on aluminum and glass (Merck & Co., Kenilworth, NJ, USA) and were visualized under UV light (254 and 365 nm) and with a Ce(SO_4_)_2_/H_2_SO_4_ solution. Reverse phase TLCs were on C18 substrate precoated on glass and were visualized under UV light (254 and 365 nm) and with a Ce(SO_4_)_2_/H_2_SO_4_ solution. Column chromatography was carried out on silica gel 230–400 mesh (EMD Chemicals Inc., Port Wentworth, GA, USA) and eluted with gradients of solvents. GC–MS data were obtained using an Agilent 6890 instrument with EI (70 eV) and Mass Selective Detector Agilent 5973N (Agilent, Santa Clara, CA, USA). The molecules were identified using the NIST mass spectral library version 1.7a. UPLC-MS data were obtained with an Agilent Technologies series 1290 infinity II instrument with the ESI ion source and QTOF Agilent Technologies G6545 model detector (Agilent, 5301 Stevens Creek Blvd, Santa Clara, CA 95051, USA). The tentative identification of the components was realized using open access web databases: FooDB, ReSpect for Phytochemicals (ReSpect), PhytoHub, and Plant Metabolic Network (PMN) and by comparison with the reported data in the literature. For the antibacterial assays, microplates were read with a Bio-Rad LightOne Illuminator (Bio-Rad Laboratories, Hercules, CA, USA).

### 3.2. Plant Material

The vegetal material was collected in Rayones (25°01′00″ N 100°05′00″ W, 906 m a.s.l.), Nuevo León, Mexico. The plant was collected and identified by the biologist Mauricio González Ferrara on 15 February 2017. A voucher reference (028029) was deposited at the herbarium of the Faculty of Biology, Universidad Autónoma de Nuevo León. The plant name has been checked in the website http://www.theplantlist.org/.

### 3.3. Extracts Preparation

Plant leaves (10.5 kg) were cleaned of residual dirt and cleaved from the pinecones, which were discarded. Then the leaves were cut into 2 to 3 cm long pieces. Part of the cut leaves (300 g) was ground fresh with distilled water (850 mL) to obtain a fresh aqueous extract. The majority of the cut leaves were then left to dry in the dark. Once dried, the vegetal material (2.29 kg) was ground first with a coffee grinder and then with a blender. Dried and ground material was macerated sequentially first with hexane (Hex) (10 L × 1, 24 h), and then with a chloroform (CHCl_3_)/methanol (MeOH) 1:1 mixture (10 L × 6, 24 h). The organic extracts were dried in vacuo whereas the aqueous extract was lyophilized. The above procedure resulted in the production of Hex (9.5 g; 0.48%), CHCl_3_/MeOH (87 g; 4.38%), and aqueous extract (8.3799 g, 2.79%), which were kept at −20 °C until use.

### 3.4. Fractionation, Isolation, Purification, and Characterization of Compounds

The Hex extract (9.0 g) was passed through a column chromatography (CC) on silica gel (180 g) and eluted with a gradient of Hex/EtOAc, obtaining a total of 226 fractions of 50 mL each. These fractions were analyzed by thin layer chromatography (TLC) under ultraviolet (UV) light, stained with Ce(SO_4_)_2_, and pooled according to their similarity into 16 fractions named from A to P. From fraction A (203.1 mg, 100% hex), a white solid (178.2 mg, 0.009%) precipitated, which was washed in cold Hex. This solid (mp 43–46 °C) was soluble in chloroform, and its nuclear magnetic resonance (NMR) spectra showed typical signals of the alkane nonacosane (1) [54]. From fractions C to F (Hex/EtOAc 98:2–92:8), a white solid (266.8 mg, 0.0143%) precipitated, which was washed in cold Hex. This solid (mp 73–75 °C) was soluble in CHCl_3_, and it was identified as hexatriacontanyl stearate (2) [55]. From fraction G (1.4490 g, Hex/EtOAc 90:10), a white solid (118 mg, 0.0015%) precipitated, which was passed through a CC on silica gel (2.36 g) eluted with a gradient of Hex/dichloromethane (DCM) yielding a white crystalline solid (29.3 mg, 0.0015%) soluble in pyridine with an mp 76–77 °C and characterized as hexacosanol (3) [56]. Fraction G was passed through four consecutive CCs on silica gel and eluted with different gradients of solvents (Hex/EtOAc x2, CHCl_3_/EtOAc x2). The last CC yielded 90 subfractions (sf), the sf 57–62 (161 mg) was then treated with a preparative TLC eluted with Hex/acetone/acetic acid (85:7.5:7.5). This procedure yielded a colorless oil (17.7 mg, 0.0009%) characterized as oleic acid (4) [57]. Fraction H (366.5 mg, Hex/EtOAc 90:10–85:15) was treated with a CC on silica gel (11 g) eluted with a gradient of Hex/EtOAc, which yielded 57 sf. Sf 4–5 (Hex/EtOAc 85:15) and yielded a white solid (273.1 mg, 0.0138%) soluble in CHCl_3_, mp 138–140 °C. It was characterized as β-sitosterol (5) [58].

The CHCl_3_/MeOH extract (85 g) was processed by CC on silica gel (2 kg) and eluted with a gradient of Hex/EtOAc/MeOH. A total of 241 fractions of 500 mL each were obtained. Fractions were analyzed by TLC under UV light and stained with Ce(SO_4_)_2_, and then pooled according to their similarity into 19 fractions (A to S). From fraction A (248.7 mg, hexane 100%), a white solid (206.2 mg, 0.0104%) precipitated, which was washed in cold Hex and dried in vacuo. This solid was soluble in CHCl_3_ and its mp was 43–45 °C (HG1). Fraction D (11.095 g, Hex/EtOAc 80:20) was treated with three consecutive CCs on silica gel eluted with different gradients of solvents (Hex/EtOAc, Hex/CHCl_3_/EtOAc x2). The last CC yielded 43 sf. From sf 32–39 (74.8 mg), a crystalline solid (55 mg, 0.0028%) precipitated, which was washed with Hex and dried in vacuo. This solid was named HG2. Fractions F-H (10.1321 g, Hex/EtOAc 50:50–30:70) were passed through a CC on silica gel (400 g) eluted with a gradient of Hex/EtOAc, which yielded 121 sf. From sf 91–110, a yellowish solid (43 mg) crystalized, which was processed through a preparative TLC (Hex/acetone 60:40) recovering 25 mg of yellowish solid. The latter was further purified by CC on silica gel (2 g) eluted with a gradient of Hex/acetone yielding 103 sf. Sf 80–102 (Hex/acetone 60:40–40:60) yielded a yellowish solid (6.6 mg, 0.0003%), soluble in acetone, with a mp 213–215 °C, and it was characterized as *p*-coumaric acid (6) [59]. Fractions F-H (Hex/EtOAc 20:80) were also chromatographed on silica gel (400 g) CC eluted with a gradient of DCM/acetone, yielding 141 sf. Sf 33–35 (Hex/EtOAc 85:15) afforded a pink solid (12.4 mg, 0.0006%), which was washed with CHCl_3_. This solid had a temperature between 80 and 82 °C, and was characterized as margaric acid (7) [54]. Fraction I (5.3493 g, EtOAc 100%) was treated with two consecutive CCs on silica gel eluted with different gradients of solvents (DCM/acetone/MeOH and CHCl_3_/MeOH). The last CC yielded 163 sf. The sf 73–126 (16.5 mg) was treated with preparative TLC and eluted with CHCl_3_/MeOH (90:10) yielding a green solid (13.5 mg), which was further purified by CC obtaining a green solid (3 mg, 0.0002%) soluble in methanol, and characterized as caffeic acid (8) [60]. From fraction J (7.4776 g, EtOAc/MeOH 70:30), a white solid (272 mg, 0.0138%) precipitated, and was washed in MeOH by sonication until it was pure. Its mp was >280 °C, and the compound was characterized as daucosterol (eleutheroside A) (9) [58]. Fractions N-S (EtOAc/MeOH 40:60 to MeOH 100%) yielded a white crystalline solid (2.7787 g, 0.14%) soluble in water, which was washed in MeOH by sonication until it was pure. This compound precipitated from the CHCl_3_/MeOH extract when it was concentrated in vacuo. Its mp was >300 °C, and it was characterized by X-ray diffraction (XRD) as potassium chloride (KCl) (10) [61].

### 3.5. Acetylation Reactions

Compounds 5 and 9 (25 mg each) were separately dissolved in pyridine (0.5 mL). Acetic anhydride (0.5 mL) was added dropwise to the stirring solutions. The reaction mixtures were left stirring overnight at room temperature (r.t.). The reaction crudes were then diluted into EtOAc (10 mL) and washed four times with a 10% HCl solution. The organic fractions were dried with anhydrous sodium sulfate and dried in vacuo to give the acetylated products. The purity of the products was assessed by TLC.

### 3.6. Methylation of Oleic Acid

Compound 4 (3.3 mg) was dissolved in toluene (0.1 mL). Then H_2_SO_4_ solution in MeOH was added dropwise to the stirring solution of the fatty acid (FA). The reaction was left stirring for 24 h at 60 °C. The reaction crude was then diluted in EtOAc (10 mL) and washed three times with NaCl (5%) and one time with NaHCO_3_ (5%) solutions. The organic phase was dried with sodium sulfate and distilled in vacuo to give the methylated product. The purity of the product was assessed by TLC.

### 3.7. GC-MS Analysis

The Hex extract and the solids HG1 and HG2 were analyzed by GC-MS using the following conditions: HP-5MS GC capillary column (30 m × 0.25 mm × 0.25 µm film thickness). Helium was used as carrier gas at a flow rate of 1.0 mL/min, the ion source and split-less injector temperatures were 230 °C and 250 °C, respectively, and the injection volume was 1–2 µL of a solution of 1 mg/mL of the extract or isolated compounds in CHCl_3_. The oven temperature was programmed from 50 °C to 285 °C at a ramping rate of 2 °C/min. The electron ionization energy was set at 70 eV, and fragments from 1 to 3000 Da were collected. The identification of the chemical constituents was carried out by comparison of the obtained mass spectra with those stored in the NIST library version 1.7a.

### 3.8. UPLC-QTOF-MS Analysis

The aqueous extract was passed through a ZOBRAX Eclipse Plus C18 HD 2.1 × 50 mm and 1.8 µm ultra-high performance liquid chromatography (UPLC) column to separate its components. The components of the extract were separated with a gradient of water and MeOH with a flux of 0.25 mL/min. The gradient started with 30% methanol and reached 100% of this solvent in 6 min, and kept this percentage for 4 min. Then, the gradient went back to 30% MeOH in 1 min. The detector was set in a positive mode in the range of acquisition between 100 and 3000 *m*/*z*. The difference of potential at the source was 3000 V, while the fragmentor voltage was 175 V. Data was extracted by molecular features. Metabolites were tentatively identified with the databases FooDB, ReSpect, PhytoHub, and Plant Metabolic Network (PMN and by comparison with the literature.

### 3.9. Bacterial Strains

The tested bacteria include nine resistant strains isolated in the University Hospital of the Universidad Autonoma de Nuevo León (Monterrey, Nuevo León, Mexico). The Gram-positive bacteria were: methicillin-resistant *Staphylococcus aureus* (14-2095), linezolid-resistant *S. epidermidis* (14-583), and vancomycin-resistant *Enterococcus faecium* (10-984). The Gram-negative bacteria were: carbapenem-resistant *Acinetobacter baumannii* (12-666), extended spectrum β-lactamase (ESBL) *Escherichia coli* (14-2081), carbapenem-resistant *Pseudomonas aeruginosa* (13-1391), oxacillin-resistant (OXA-48) *Klebsiella pneumoniae*, ESBL *K. pneumoniae* (17-1692), and New Delhi metallo-β-lactamase 1 (NDM-1+) *K. pneumoniae* (14-3335).

### 3.10. Antibacterial Activity

The minimum inhibitory concentration (MIC) of the extracts, fractions, and pure compounds were determined in duplicate by the micro-dilution broth method in 96-well microplates [62]. The aqueous extract was dissolved in distilled water, while organic extracts and pure compounds were dissolved in dimethyl sulfoxide (DMSO). The solutions were then diluted in Mueller-Hinton broth (Difco, Detroit, MI, USA), in order to achieve concentrations ranging from 500 to 7.81 µg/mL for extracts and 200 to 3.125 µg/mL for pure compounds. The range of concentrations used for dimethyl sulfoxide (DMSO) was from 6% to 0.1% (*v*/*v*) and this solution was used as a negative control. The inoculum was adjusted to a concentration of 5 × 10^5^ CFU/mL, according to the National Committee for Clinical Laboratory Standard (NCCLS) guidelines (CLSI, 2015). Levofloxacin was used as a positive control for resistant strains and its concentrations ranged between 200 and 3.125 µg/mL. Controls of sterility of the extracts, control of the inoculum, and control of the DMSO were performed. The 96-well microplates were sealed and incubated aerobically at 37 °C for 24 h with the exception of *S. epidermidis*, which was incubated for 48 h. After the incubation, the turbidity or bottom deposition was visually evaluated to determine the microorganism viability. The MIC values were determined as the lowest concentration of extracts or pure compounds able to inhibit the microorganism growth.

## 4. Conclusions

This is the first report of secondary metabolites of *H. glomerata* and is the first in the genus *Hechtia*. There is a need for bioassay guided studies of this plant with sensitive bacteria in order to explain its use in Mexican traditional medicine. Additionally, it is necessary to isolate the active compounds from Hex and aqueous extracts to identify a good candidate for treating resistant bacterial infections.

## Figures and Tables

**Table 1:** GC-MS analysis of Hex extract, compounds **1**-**5**, HG1 and HG2 from *H. glomerata*.

Compound	R_T_ (Min)	% Area	
		Hex	HG1, HG2
Octanal	10.580	0.09	-
Nonanal	16.144	0.11	-
1-Dodecene	21.431	-	0.72
2-(*E*)-Decenal	25.918	0.15	-
1-Tetradecene	34.148	-	2.37
1-Hexadecene	45.946	-	1.37
Unknown	50.143	-	0.02
12-Methyltetradecanic acid (C14:0, anteiso)	56.817	0.36	-
Pentadecanoic acid (C15:0)	58.400	2.16	-
Isopropyl myristate	58.419	0.03	-
Phytone	59.332	2.65	-
*n*-Nonadecane	62.006	0.16	-
Palmitoleic acid (C16:1, ∆9)	62.183	0.68	-
Trachylobane	65.054	0.11	-
Palmitic acid (C16:0)	65.881	4.25	-
5-(*E*)-Eicosene	66.440	0.15	-
14-Methylpalmitic acid (C17:0, iso)	66.748	2.85	-
*n*-Eicosane	66.755	0.29	-
2-Hexyl-cyclopropaneoctanoic acid	67.261	0.83	-
Kaur-16-ene	67.497	0.16	-
Margaric acid (C18:0)	68.036	0.75	-
*Trans*-vaccenic acid (C18:1, ∆11)	69.632	0.53	-
*n*-Heneicosane	71.366	3.04	7.34
Phytol	71.918	1.72	-
Linoleic acid (C18:2, ∆9,12)	73.842	8.35	-
Ethyl linoleate	74.066	5.44	-
Ethyl oleate	74.368	1.72	-
Nonadecenoic acid (C19:1, ∆13)	74.374	1.64	-
Oleic acid (C18:1, ∆9)	74.861	6.72	-
*n*-Docosane	75.701	0.64	1.46
Gondonic acid (C20:1, ∆11)	79.872	0.74	-
*n*-Tricosane	80.162	-	6.16
Cyclotetracosane	83.689	0.14	-
*n*-Tetracosane	84.004	0.55	1.12
12-(*Z*)-Pentacosene	87.591	0.07	-
*n*-Pentacosane	88.005	2.40	3.82
Unknown	89.528	3.23	1.24
9-Hexacosene	91.381	0.63	2.27
*n*-Hexacosane	91.676	0.64	1.28
12-(*Z*)-heptacosene	95.033	0.50	-
*n*-Heptacosane	95.243	1.05	6.63
Unknown	96.747	3.24	-
Cycloocatacosane	98.909	-	12.78
*n*-Octacosane	99.040	-	3.00
Nonacosanol	101.871	0.19	-
*n*-Nonacosane	102.219	4.19	28.63
1-Triacontanol	105.313	-	1.70
*n*-Triacontane	105.497	0.33	2.56
Hentriacontane	108.545	2.13	8.18
Ergosterol	110.896	0.24	-
Campesterol	111.573	5.38	-
Stigmasterol	112.512	1.37	-
Hentriacontanol	112.867	0.14	-
1,30-Triacontanediol	113.175	2.78	-
β-Sitosterol	114.607	17.97	-
Dotriacontenol	114.969	-	7.34
(3β,5α)-Ergostanol	115.921	0.49	-
Stigmast-4-en-3-one	118.233	6.02	-

R_T_: Retention time. The compounds were identified using the National Institute of Standards and Technology (NIST) mass spectral database (version 1.7a).

**Table 2:** Components identified from the aqueous extract of *H. glomerata* by UPLC-QTOF-MS.

Compound	R_T_ (min)	Molecular Formula	Experimental *m*/*z*	Error (ppm)	Means of Identification
3,8-Diglucosyldiosmetin	1.967	C_28_H_32_O_16_	625.167	0.93	Foodb
5,7-Dihydroxy-6-methoxyisoflavone 7-*O*-rhamnoside	2.986	C_22_H_22_O_9_	431.1264	0.73	Foodb
Unknown	3.962	-	412.259	-	-
Delphinidin 3-sophoroside 5-glucoside	4.771	C_33_H_41_O_22_^+^	789.67	−4.61	Foodb
Phytyl monophosphate	5.023	C_20_H_39_O_4_P	375.1947	7.23	Foodb
Catechin 4′-methyl ether	5.543	C_16_H_16_O_6_	305.095	0.73	Foodb
6-tuliposide A	5.945	C_11_H_18_O_8_	279.1519	−4.45	Foodb
Daucosterol	6.152	C_35_H_60_O_6_	577.86	−4.24	[33]
Peonidin	6.346	C_18_H_20_O_4_^+^	301.1339	−6.27	Foodb
Unknown	6.356	-	325.130	-	-
Guanosine pentaphosphate	6.377	C_10_H_12_N_5_O_17_P_4_	599.123	1.63	PMN
Epicatechin 3-*O*-gallate	6.421	C_22_H_18_O_10_	443.3262	−2.29	Foodb
Chlorogenoquinone	6.442	C_18_H_24_O_7_	353.1502	−6.35	Foodb
Tetramethylquercetin	6.474	C_19_H_18_O_7_	359.3165	−2.03	Foodb
Kaempferol 3-*O*-rutinoside	6.573	C_27_H_30_O_15_	594.159	1.01	[33]
Luteolin 7-(2′′-apiosylglucoside)	6.643	C_26_H_28_O_15_	581.143	0.73	[33]
Ergosterol endoperoxide	6.714	C_28_H_44_O_3_	429.311	2.53	[33]
1, 28-Dicaffeoyloctacosanediol	6.761	C_46_H_70_O_8_	751.5143	−0.01	Foodb
1,26-Hexacosanediol diferulate	6.761	C_46_H_70_O_8_	751.507	0.73	Foodb
Proanthocyanin monogallate	6.761	C_36_H_56_O_15_	729.829	−6.79	Respect
Epicatechin 3-*O*-(3-*O*-methylgallate)	7.160	C_23_H_20_O_10_	457.2491	−1.36	Foodb
Piceid	7.26	C_20_H_22_O_8_	391.2785	−1.40	PhytoHub
Cyanidin-3-*O*-(2′′-*O*-β-xylopyranosyl-β-glucopyranoside)-5-*O*-β-glucopyranoside	7.274	C_32_H_39_O_20_^+^	745.625	1.50	Respect
Tricin	7.300	C_17_H_14_O_7_	331.1676	−8.64	Foodb
Kaempferol 3-triglucoside-7-rhamnoside-p-coumaroyl	7.345	C_48_H_56_O_27_	1065.309	−0.90	Respect
Spinacetin 3-*O*-glucosyl-(1–>6)-[apiosyl(1–>2)]-glucoside	7.348	C_34_H_42_O_22_	803.684	−4.60	PhytoHub
Hexosyl-hexosyl-soyasapogenol E	7.348	C_42_H_68_O_13_	780.47	-	Phytohub
Oleanolic acid 3-*O*-glucose(1′′–>3′)arabinose	7.348	C_41_H_66_O_12_	751.460	0.27	Foodb
3-*O*-Rutinosyl-3′-*O*-glucopyranosyl cyanidin	7.382	C_33_H_41_O_20_^+^	757.67	−4.51	[33]
Quercetin-3-Galactoside-6′′-Rhamnoside-3′’’-Rhamnoside	7.386	C_34_H_42_O_20_	771.700	−0.47	Respect
3,7,3′-*O*-Triglucopyranosyl-dephinidine	7.386	C_33_H_41_O_22_^+^	789.570	1.00	[33]
Hederagenin 3-*O*-hexose-pentose	7.391	C_41_H_66_O_13_	766.430	2.00	Respect
Prenyl caffeate	7.413	C_14_H_16_O_4_	249.121	0.24	Foodb, PhytoHub
Peonidin 3-sophoroside	7.555	C_28_H_33_O_16_^+^	625.177	0.01	Foodb
Unknown	7.776	-	348.486	-	-
Trihydroxycinnamoylquinic acid	7.801	C_16_H_18_O_10_	371.200	-1.10	Respect
Cinnamtannin B1	7.802	C_45_H_36_O_18_	865.6147	-4.17	Phytohub
Galloylquinic acid isomer	7.827	C_38_H_40_O_19_	801.220	-1.06	Foodb
Agavoside B	7.837	C_34_H_42_O_19_	755.686	-2.65	Foodb
Cyanidin 3,5,3′-tri-*O*-glycoside	7.838	C_33_H_41_O_21_^+^	774.595	-3.74	[33]
Unknown	7.874	-	674.579	-	-
Luteolin 7-*O*-(2-apiosyl-6-malonyl)-glucoside	7.876	C_29_H_30_O_18_	667.538	-3.87	Foodb
Petunidin 3-*O*-(6-*O*-acetyl)-glucoside	7.901	C_24_H_25_O_13_^+^	522.589	-4.53	Foodb
Piceatannol 4′-glucoside	8.022	C_20_H_22_O_9_	407.129	0.51	Foodb
Peonidin 3-rhamnoside	8.185	C_22_H_23_O_10_^+^	447.361	-2.32	Foodb
Myricetin 3,3′-digalactoside	8.228	C_27_H_30_O_18_	643.519	-3.69	[33]
Aloesol 7-glucoside	8.248	C_19_H_24_O_9_	397.1759	-2.62	Foodb
Delphinidin 3-rutinoside 5-glucoside	8.299	C_33_H_41_O_21_^+^	774.669	-4.48	Foodb
Myricitrin glucoside	8.302	C_21_H_20_O_12_	465.0954	0.74	[33]
Luteolin 7-*O*-diglucuronide	8.341	C_27_H_26_O_18_	639.485	−3.65	foodb
Unknown	8.856	-	426.729	-	-
3-*O*-Glucopyranosyl-6,3′,5′-trimethoxy-3,5,7,4′-tetrahydroxyflavone	9.401	C_24_H_26_O_14_	539.460	1.10	[33]
Zeaxanthin	9.401	C_40_H_56_O_2_	569.881	−4.46	PMN
Ananaflavoside B	10.158	C_25_H_28_O_13_	537.3871	1.03	[33]
Isochlorogenic acid B	10.158	C_26_H_26_O_11_	515.483	−3.35	PhytoHub

**Table 3:** Activity of *H. glomerata* leaves extracts, major isolated compounds, and their semisynthetic derivatives against resistant bacteria.

Extracts	MIC (µg/mL)								
	*S. aureus* methicillin	*S. epidermidis* Linezolid	*E. faecium* Vancomycin	*A. baumannii* Carbapenems	*P. aeruginosa* Carbapenems	*E. coli* ESLB	*K. Pneumoniae* NDM-1 +	*K. pneumoniae* ESBL	*K. Pneumoniae* Oxacillin
Hex	>500	>500	>500	>500	>500	**500**	**500**	**500**	500
CHCl_3_/MeOH	>500	>500	>500	>500	>500	>500	>500	>500	>500
Aqueous	>500	>500	>500	>500	>500	**500**	**500**	**500**	500
**Compounds**									
5	>200	**200**	**200**	**200**	**200**	>200	**200**	>200	>200
5a	>200	**200**	**200**	**100**	**200**	**200**	>200	**200**	>200
6	>200	>200	>200	>200	>200	>200	>200	>200	>200
8	>200	**200**	**200**	>200	>200	>200	>200	>200	>200
9	>200	**200**	**200**	**200**	**200**	>200	>200	>200	>200
9a	>200	>200	**200**	**200**	**200**	>200	>200	>200	>200
Levofloxacin	12.5	6.25	12.5	12.5	0.78	25	>50	0.78	12.5

β-sitosterol (5), β-sitosteryl acetate (5a), p-coumaric acid (6), caffeic acid (8), daucosterol (9), daucosteryl acetate (9a). The samples with MIC values ≤ 500 μg/mL are written in bold.
